# Eliminating all circulating vaccine-derived poliovirus: a prerequisite to declaring global polio eradication

**DOI:** 10.1093/inthealth/ihac068

**Published:** 2022-10-22

**Authors:** David N Durrheim

**Affiliations:** University of Newcastle, Newcastle, NSW, Australia

Eradication is an all-or-nothing affair. The aspirational goal set by the World Health Assembly in 1988 of eradicating poliomyelitis by 2000 was not achieved.^1^ A 99.9% reduction in cases is a wonderful outcome for the estimated 1.5 million people whose lives have been saved and the 16 million people who have been spared lifelong disability since the Global Polio Eradication Initiative was launched, but it is an eradication failure! The massive sustained global investment that saw improved immunisation coverage and enhanced surveillance in most countries did result in the reporting of the last human cases of wild poliovirus type 2 in 1999 and type 3 in 2012. Certification of wild poliovirus type 2 and type 3 eradication occurred in 2015 and 2019, respectively.^2^

It is thus tempting to consider eradication of poliomyelitis two-thirds complete, with wild poliovirus type 1 in Pakistan and Afghanistan being the final hurdle to finishing the ‘polio end-game’. But this is sadly not the case. The protracted eradication efforts and perpetually underperforming vaccination programmes using oral live attenuated Sabin poliovirus vaccine (OPV) have birthed calamitous outbreaks of types 1, 2 and 3 circulating vaccine-derived poliovirus (cVDPV) in countries in every World Health Organization region since the first recorded cVDPV outbreak in Haiti in 2020.^3^ These viral strains, with reverted neurovirulence, can produce paralysis similar to wild poliovirus. It has been recognised for more than a decade that sustained person-to-person transmission can occur in any country, including high-income countries, where pockets of underimmunised individuals exist.^4^

The ‘big switch’ from trivalent OPV (TOPV; covering types 1, 2 and 3) to bivalent oral polio vaccine (BOPV; covering types 1 and 3), while introducing at least one dose of inactivated poliovirus vaccine (IPV) for every child, was necessary to reduce the ongoing risk of cVDPV2 outbreaks. Unfortunately the tardy rollout of IPV and outbreak response campaigns using monovalent OPV type 2 have increased the risk of cVDPV outbreaks.^5^ The perverse impacts of the coronavirus disease 2019 pandemic on childhood immunisation coverage, surveillance quality and postponed supplementary immunisation campaigns are starkly illustrated in languishing national and subnational acute flaccid paralysis surveillance performance and the disquieting increase in cVDPV outbreaks in the past 2 years.^1,6^ The unprecedented surge in reported confirmed human infections and increasing number of countries with cVDPV detections, either human infections or through environmental surveillance, is cause for great concern (Table 1).

**Table 1.** Global cVDPV types 1, 2 and 3 by countries affected and total cases detected, 2016–2021^a^

	cVDPV1 countries with	cVDPV2 countries with	cVDPV3 countries with	Total number of human
Year	detections^b^ (cases), n (%)	detections^b^ (cases), n (%)	detections^b^ (cases), n (%)	cases^c^
2016	1 (8)	2 (5)	0 (0)	13
2017	0 (0)	3 (181)	0 (0)	181
2018	2 (34)	7 (145)	1 (9)	188
2019	5 (22)	19 (543)	0 (0)	565
2020	5 (35)	30 (1367)	1 (1)	1403
2021	2 (41)	31 (1016)	3 (0)	1057
Total cases^c^	140	3257	10	3407

^a^Based on data reported by the Global Polio Eradication Initiative (https://polioeradication.org/polio-today/polio-now/this-week).

^b^Total countries with either a confirmed human case or detection of cVDPV by environmental surveillance.

^c^Includes all human cases (both acute flaccid paralysis and other).

We must not bury our heads in the sand. Novel, genetically more stable oral polio vaccines used under emergency authorisation for cVDPV2 outbreak response provide a glimmer of hope, but this alone is not enough.^7^ The only solution that will permanently rid the world of the cVDPV threat is achieving high universal childhood immunisation coverage. All countries using OPV in their national immunisation programmes should provide timely vaccination (three doses of BOPV and two doses of IPV) to every child.^8^ Eliminating the emergence and spread of cVPDVs will be an excellent marker of achieving the worthy Immunisation Agenda 2030 goal of ‘not leaving anyone behind’.^9^
